# Understanding Cardiology Practitioners’ Interpretations of Electrocardiograms: An Eye-Tracking Study

**DOI:** 10.2196/34058

**Published:** 2022-02-09

**Authors:** Mohammed Tahri Sqalli, Dena Al-Thani, Mohamed B Elshazly, Mohammed Al-Hijji, Alaa Alahmadi, Yahya Sqalli Houssaini

**Affiliations:** 1 Information and Computing Technology Division College of Science and Engineering Hamad Bin Khalifa University Doha Qatar; 2 Department of Cardiac Electrophysiology Cleveland Clinic Cleveland, OH United States; 3 Johns Hopkins Ciccarone Center Heart and Vascular Institute Johns Hopkins Medicine Baltimore, MD United States; 4 Interventional & Structural Cardiology Division Heart Hospital Hamad Medical Corporation Doha Qatar; 5 Weill Cornell Medical College Cornell University Doha Qatar; 6 College of Computer Science and Engineering Taibah University Yanbu Saudi Arabia; 7 Department of Medicine Faculty of Medecine and Pharmacy Mohammed V University Rabat Morocco

**Keywords:** eye tracking, electrocardiogram, ECG interpretation, cardiology practitioners, human-computer interaction, cardiology, ECG

## Abstract

**Background:**

Visual expertise refers to advanced visual skills demonstrated when performing domain-specific visual tasks. Prior research has emphasized the fact that medical experts rely on such perceptual pattern-recognition skills when interpreting medical images, particularly in the field of electrocardiogram (ECG) interpretation. Analyzing and modeling cardiology practitioners’ visual behavior across different levels of expertise in the health care sector is crucial. Namely, understanding such acquirable visual skills may help train less experienced clinicians to interpret ECGs accurately.

**Objective:**

This study aims to quantify and analyze through the use of eye-tracking technology differences in the visual behavior and methodological practices for different expertise levels of cardiology practitioners such as medical students, cardiology nurses, technicians, fellows, and consultants when interpreting several types of ECGs.

**Methods:**

A total of 63 participants with different levels of clinical expertise took part in an eye-tracking study that consisted of interpreting 10 ECGs with different cardiac abnormalities. A counterbalanced within-subjects design was used with one independent variable consisting of the expertise level of the cardiology practitioners and two dependent variables of eye-tracking metrics (fixations count and fixation revisitations). The eye movements data revealed by specific visual behaviors were analyzed according to the accuracy of interpretation and the frequency with which interpreters visited different parts/leads on a standard 12-lead ECG. In addition, the median and SD in the IQR for the fixations count and the mean and SD for the ECG lead revisitations were calculated.

**Results:**

Accuracy of interpretation ranged between 98% among consultants, 87% among fellows, 70% among technicians, 63% among nurses, and finally 52% among medical students. The results of the eye fixations count, and eye fixation revisitations indicate that the less experienced cardiology practitioners need to interpret several ECG leads more carefully before making any decision. However, more experienced cardiology practitioners rely on their skills to recognize the visual signal patterns of different cardiac abnormalities, providing an accurate ECG interpretation.

**Conclusions:**

The results show that visual expertise for ECG interpretation is linked to the practitioner’s role within the health care system and the number of years of practical experience interpreting ECGs. Cardiology practitioners focus on different ECG leads and different waveform abnormalities according to their role in the health care sector and their expertise levels.

## Introduction

Visual expertise refers to advanced visual skills demonstrated when executing domain-specific visual tasks [1]. Understanding health care practitioners’ visual expertise is crucial in clarifying how to best acquire accurate interpretations of medical images. Visual expertise may be gained through clinical experience, active learning, or teaching. In the context of this study, we shed light on the visual skill of electrocardiogram (ECG) interpretation and, more specifically, on the methodological practices used by cardiology practitioners when conducting visual 12-lead ECG interpretations [2,3]. State-of-the-art visual expertise research has primarily focused on the medical image interpretations of x-rays, mammograms [4,5], and computed tomography and magnetic resonance imaging scans [6,7]. This study is one of few others [3,8-11] that explores how visual expertise contributes to the accuracy of ECG interpretation.

The importance of conducting this study stems from the fact that the ECG is one of the most used medical tests in modern medicine, reaching over 300 million ECGs done annually in the United States alone [12]. In addition, accurate interpretation remains a challenge since there appears to be significant erroneous interpretation rates among nurses, residents, and fellows [3]. Another challenge facing ECG interpreters is the variation in the interpretation procedures and guidelines across different regulating bodies and institutions [13]. Thus, there is a need for additional insights to establish better educational and working practices that suit the different expertise levels of cardiology practitioners to acquire the essential skills for accurately interpreting the ECG. Previous studies focusing on visual expertise in ECG interpretation have mainly restricted their emphasis to the visual aspect of interpretation. Those studies focus primarily on generating eye movement heat maps and statistical data [3,8-11]. However, those same studies lack a discussion on the link between the observational visual behavior of the interpreter and the ECG diagnosis strategy, a critical element that contributes to an accurate ECG interpretation [3].

This study extends the results of our initial work [14-19] under the theme of where does the use of technology fall in the medical landscape. More precisely, one of our studies [14] is aimed at understanding how medical students start to acquire the skill of ECG interpretation. This study focuses more on the cardiology practitioners who interpret ECGs as part of their daily clinical practice. The essence of this study is to pave the way for understanding the link between observational visual behavior and final ECG diagnostics as an element of visual expertise for ECG interpretation. The objective of our study is to quantify and analyze, using eye-tracking technology, differences in cardiology practitioners’ visual expertise in ECG interpretation. This quantification is done considering the number of years of practitioners’ clinical experience as they advance their medical careers. To reach this objective, we identify eye-tracking metrics that serve this purpose and provide insights into interpretation methodological strategies underpinning accurate ECG interpretation. We then conduct an eye-tracking study with five different categories of cardiology practitioners with different expertise levels. The quantitative results provide insights into interpretation methodological strategies underpinning accurate ECG interpretation, which varies according to the number of years of practical experience in ECG interpretation. Finally, ECG interpretation trends among the pool of participants are unveiled by creating matches between eye fixation heat maps and other eye-tracking metrics.‬‬‬‬

## Methods

### Hypotheses

Related works focusing on the relationship between visual expertise, ECG interpretation [3,8-11], and other clinical fields [4-7] requiring medical images interpretation were taken into consideration before creating the following hypotheses. The eye-tracking study by Davies et al [10] especially inspired our second and third hypothesis as the authors noted that experienced interpreters adopt a duel processing model of ECG interpretation. Additionally, the study by Wu et al [3] also emphasized this nuance among different categories of medical practitioners. The following are our three hypotheses:

1. There exists a significant quantifiable difference in the accuracy of the interpretation of each expertise level category of participants as they gain more years of experience.
2. There is a significant correlation between the number of years of participant’s experience, depicted by their cardiology practitioner roles, and their fixations’ behavior around specific areas of the ECG, demonstrated by the fixations count.
3. There is a significant correlation between the number of years of participant’s experience, depicted by their cardiology practitioner roles, and their eye movement transition frequency between different parts/leads on the standard 12-lead ECG, demonstrated by fixation revisitations.

### Study Design

The conducted study uses eye-tracking technology to quantify and understand differences in human visual behavior during ECG interpretation. With different clinical roles in the health care sector, recruited participants were tasked with interpreting 10 ECGs with different types of cardiac abnormalities. During their interpretation, their eye movements were recorded using an eye tracker and the collected eye movements data was analyzed quantitatively. Participants were also tasked with selecting their final diagnosis for each ECG from among four available choices or writing down a diagnosis other than those proposed. The choices for each ECG are available in Multimedia Appendix 1.

The experiment used a counterbalanced within-subjects design with the following one independent variable: the expertise level of the cardiology practitioner. This can be quantified as a categorical variable based on the number of years of ECG interpretation experience, as described in Table 1. Medical practitioners may also be placed in one of the clinical categories/roles highlighted in Table 1.

Two measured dependent eye-tracking variables were expected to change when the independent variable changed. These two variables are measured according to our definition of grid-based areas of interest (AOIs). A sample grid-based AOI applied to the normal sinus rhythm ECG can be referred to in Multimedia Appendix 2. Our explanation behind our choice for the grid-based AOIs can be found in our previous work [14]. The following are the two dependent variables:‬‬‬‬

- The average fixations count for each ECG lead for each category of participant
- The average fixation revisitations for each ECG lead for each category of participant

The experiment also had one control variable. The time given for each participant to look at each ECG was limited to 30 seconds. This time limit allowed for all categories of participants to be held to the same standards in terms of the amount of time given for them to analyze each case. This time limit was chosen by consulting the cardiology consultant and professor involved in designing this experiment. The time is also supported by studies investigating the choice of this parameter within different categories of medical practitioners such as medical students and consultants. The time allowed for scanning an ECG was found to have no statistically significant effect on the result of the diagnosis [20]. It was also found that there is a negative correlation between the duration spent looking at an ECG and the accuracy of the final interpretation provided [21].

**Table 1:** Corresponding variables to each hypothesis.

Hypothesis	Independent variable	Dependent variable
Hypothesis 1	Years of experience	Accuracy of interpretation
Hypothesis 2	Years of experience	Fixations count
Hypothesis 3	Years of experience	Fixation revisitations

### Participants

Table 1 summarizes the demographics of the participants included in this study. A total of 63 participants with varying ECG interpretation expertise were recruited from a university campus and a cardiac hospital. Participants were recruited based on their medical category represented by their job title/role in clinical practice. The mean age was 28 (SD 4) years. In addition, participants were asked to provide an approximation of their years of work experience in ECG interpretation. The medical categories are defined as follows:

- Junior medical students: those in a preclinical curriculum
- Senior medical students: those in a clinical curriculum
- Nurses: nurses either serving in the catheterization laboratory or the cardiac care unit
- Technicians: cardiovascular technologists working in a cardiac catheterization laboratory
- Fellows: physicians undergoing postgraduate training in cardiology
- Cardiology consultants: board-certified independent cardiology practitioners

### Stimuli Design

The ECG stimuli were acquired from the collection belonging to the cardiology consultant involved in designing the experiment. Since the study is motivated by quantifying visual behavior across different expertise levels of different health care practitioners, we selected ECGs commonly encountered by all those categories in their day-to-day medical practice [22]. The ECGs sampled are defined in Multimedia Appendix 3. We limited our selection to 10 representative ECG cases.

### Apparatus

A Tobii Pro X2-60 eye tracker and iMotions version 8.1 software [23] were used to track eye movements with a frequency of 60 Hz (±1 Hz). In addition, keyboard presses and mouse input were recorded to register the participants’ responses showing their final diagnosis for each ECG. The study was conducted on a 25-inch diagonal laptop monitor with a resolution of 1366 by 768 pixels.

### Ethics

This study received institutional review board approval from the ethical board of both the Qatar Biomedical Research Institute at Hamad bin Khalifa University [24] under the research protocol number QBRI-IRB-2020-01-009 and the Hamad Medical Corporation under the research protocol number MRC-02-20-714. Approvals were granted before the start of the experiment. Institutional review board approval guarantees that all study methods were conducted following the guidelines and recommendations of international regulatory agencies [24].

### Analysis

#### Hypotheses Testing Methods

The three hypotheses regarding participants’ visual behavior toward ECG interpretation were tested as follows.

##### Analysis Method for Testing Hypothesis 1

To test the first hypothesis, interpretations were assessed for participants’ accuracy by determining if they chose the correct ECG diagnosis from among the four offered choices. Analyzing the participants’ accuracy of interpretation scores using the Cramér V statistical test contributed toward constructing a clear understanding of how much the interpreters understood the ECG signals and its waveform abnormalities presented to them throughout the 10 ECG cases.

##### Analysis Method for Testing Hypothesis 2

To test the second hypothesis, interpretations were assessed for the frequency with which the participants fixated on ECG images. This assessment was done by comparing eye movement behavior for the five categories. Eye movement was quantified using a median fixations count for each participant. A prior study showed that the average duration for one fixation ranged from 150 to 300 milliseconds [25]. Although the average fixation duration span has a fixed range, the fixation count provides a more accurate depiction of the interpreter’s attention. The fixations count number represents the interpreter’s engagement with different ECG leads suggesting that the greater the median fixation duration, the greater the level of engagement [3].

##### Analysis Method for Testing Hypothesis 3

To test the third hypothesis, interpretations were assessed for the frequency with which the interpreters revisit different areas, or leads, in the ECG. This was done by comparing each participant’s average ECG lead revisitation among the five categories. A *lead revisitation* is defined as the interpreter fixating again on a particular lead after visiting it previously.

## Results

### Results for Testing Hypothesis 1

**Figure 1:**
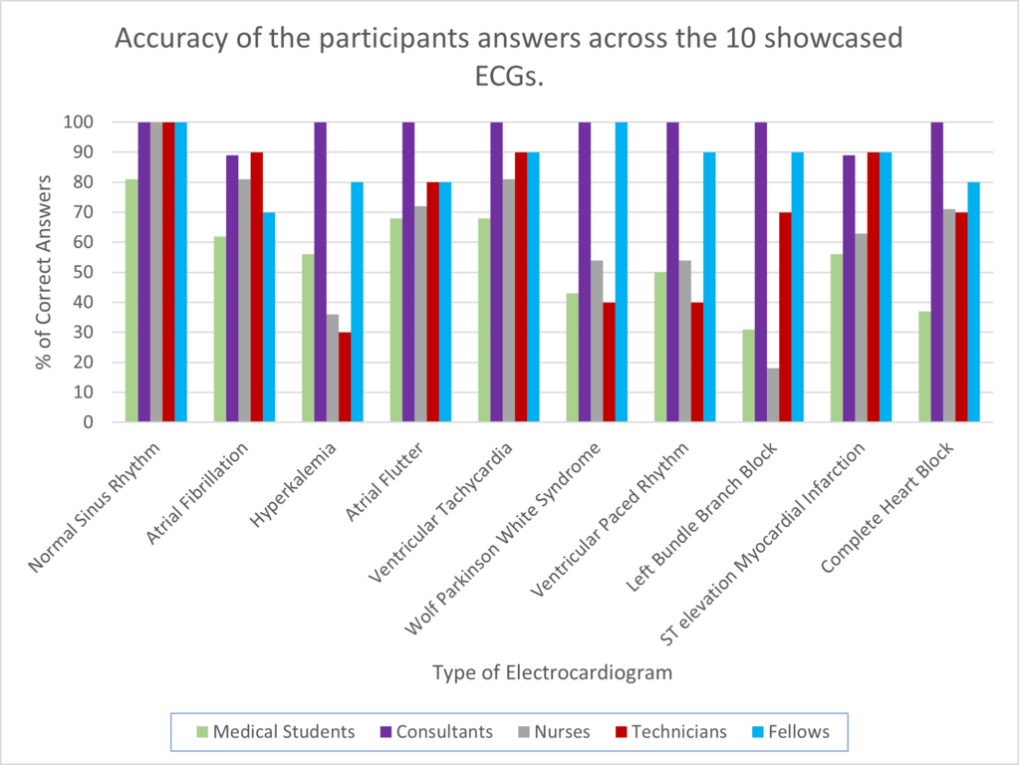
Accuracy of the participants’ answers across the 10 showcased ECGs. ECG: electrocardiogram.

Figure 1 summarizes the accuracy of the participants’ answers across the 10 showcased ECGs. Consultants are the most accurate interpreters, with an accuracy percentage of 97.8%. Fellows are the second most accurate, with an overall accuracy of 87%, followed by technicians with an accuracy of 70%. Nurses were the least accurate of those with working experience, with an accuracy of 63%. Finally, medical students were overall the least accurate category with an accuracy of 52.2%. A chi-square test was conducted since the interpretation response data is dichotomous. The obtained *P* value was .02, which shows that there is a statistically significant difference in interpretation accuracy between the five categories. To calculate the effect size of the chi-square independence test, we used the Cramér V, providing a value of 0.36 that indicates a weak association between the categories. Although some ECGs were easy to interpret correctly (eg, the normal sinus rhythm), other ECGs, including the ventricular paced rhythm and the left bundle branch block, were harder to interpret correctly.

### Results for Testing Hypothesis 2

Table 2 summarizes the median and SD from the IQR for the total fixations count per participant for all leads in the ECG. The fixations data follows a nonnormal, left-skewed distribution. Total fixation counts were calculated for the 10 ECGs for 300 seconds. Table 2 also includes the median and SD from the IQR for the fixation count per lead across all 10 ECGs. Consultants are the category with the lowest number of fixations, while medical students have the highest number of fixations. Applying the Kruskal-Wallis test to the total fixations count data resulted in a *P* value of .03, which showed there is a statistically significant difference in participants’ fixation count.

**Table 2:** Demographics for the participants included in the eye-tracking study.

Feature and demographics		Participants, n
**Medical category**		
	Medical students (junior year)	9
	Medical students (senior year)	10
	Fellows	11
	Technicians	10
	Nurses	14
	Consultants	9
**Age (years)**		
	20-23	10
	23-25	9
	26-30	21
	30-35	11
	35-45	12
**Gender**		
	Male	51
	Female	12
**Years of experience**		
	0 years	10
	1 year	9
	2-5 years	15
	5-10 years	22
	≥15 years	7

### Results for Testing Hypothesis 3

Table 3 summarizes the median total fixations count per participant and median fixation count per lead per participant, while Table 4 summarizes the mean and SD of the ECG lead revisitations for each category of participants. The ECG lead revisitations data follows a normal distribution. On average, technicians are the category of participants with the highest number of revisitations for each lead with an average of 3.61 revisitations, while consultants are the category that revisits a lead the least with an average of 2.01 revisitations. However, based on the SD results, variation among participants in the same category was the highest among nurses and the lowest among technicians. A one-way analysis of variance test was applied to the data, showing an *F* value of 30.56, which is larger than the critical *F* value (2.36). We measured the effect size using the Eta squared formula and the result is η^2^=0.36.

**Table 3:** Median total fixations count per participant and median fixation count per lead per participant.

Category	Fixations count per participant (for all ECGs^a^)			Fixation count per lead for each ECG	
	Median	SD from the IQR	Median		SD from the IQR
Medical students	2829	1411	9.93		5.01
Technicians	2535	301	10.83		1.27
Nurses	2444	1031	9.49		3.96
Fellows	2135	579	9.12		2.52
Consultants	1385	794	6.57		3.95

^a^ECG: electrocardiogram.

**Table 4:** Average electrocardiogram (ECG) lead revisitation per participant for every category.

Category	ECG lead revisitation per participant	
	Mean (μ)	SD (σ)
Technician	3.61	0.06
Nurse	3.25	1.60
Medical students	2.90	0.85
Fellow	2.55	0.67
Consultant	2.01	0.98

## Discussion

### Insights From the Eye-Tracking Results

The results indicate that the interpreter’s expertise, revealed by the number of years of work experience in ECG interpretation, is the primary influence for both the accuracy of ECG interpretation and the acquired visual expertise strategies. Through the analysis of the three hypotheses, three main findings were confirmed.

First, the accuracy of ECG interpretation correlates with the expertise level of the participant. The results confirm the first hypothesis by indicating that consultants are the category with the most accurate interpretations, while medical students are the category with the least accurate interpretations. In between these two extremes are nurses, technicians, and fellows.

Second, as expertise for participants increases, participants’ fixations count on ECG signal waveform abnormalities decreases. This finding translates into participants fixating on the overall ECG for less time while not compromising the accuracy of the interpretation. This finding confirms the second hypothesis.

**Figure 2:**
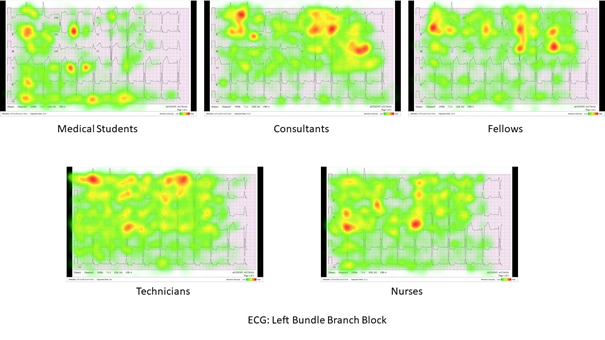
Sample aggregate heat maps showing differences in fixation distribution across the left bundle branch block ECG between the studied categories. ECG: electrocardiogram.

**Figure 3:**
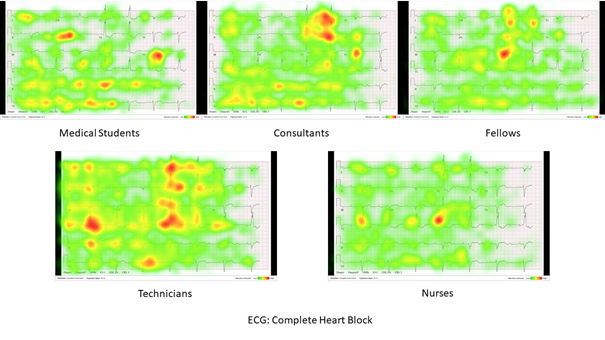
Sample aggregate heat maps showing differences in fixation distribution across the complete heart block ECG between the studied categories. ECG: electrocardiogram.

Third, the results for testing hypothesis 3 confirm that medical practitioners observe and focus on certain ECG leads and waveform abnormalities according to their role in the health care sector and their expertise level. Figures 2 and 3 show sample aggregate heat maps for the differences in fixation distribution across the left bundle branch block and the complete heart block ECGs between the studied categories. The results from the third assessment aiming to confirm the third hypothesis indicate that both consultants and fellows target their fixations on specific leads to identify the correct ECG diagnosis. This finding was also confirmed by looking at the heat maps for different categories across different ECGs other than the ones in Figures 2 and 3. However, nurses and technicians thoroughly interpreted the ECGs systematically by scanning through all 12 leads and primarily looking at abnormalities in the ST segment and wide QRS complex. This finding explains the high number of fixations per lead measured by the fixations count. This behavior may be because nurses are usually not extensively trained to interpret the ECG the way cardiologists do for a diagnosis but to ensure that signs of imminent heart attacks are not missed. The heat maps of medical students indicate that they randomly fixate on the waveform abnormalities that they first perceive and then continue to transition from one lead to another until the 30-second time limit is over. This finding justifies medical students having the highest number of fixations per ECG case. Heat maps for all the categories of interpreters and all the ECGs can be found in Multimedia Appendix 4.

### Conclusion

#### Concluding Remarks

This paper presents a quantitative analysis of the ECG interpretation visual behavior of different health care practitioners. These health care practitioners belonged to five different categories: medical students, nurses, technicians, fellows, and consultants. Eye-tracking data for these categories were collected while they each interpreted a total of 10 ECGs. Specific eye-tracking metrics such as fixations count and fixation revisitations were quantitatively analyzed for each lead in the standard 12-lead ECG and across all ECGs. This analysis was done to meet the objective of quantifying, using eye tracking, medical practitioners’ visual expertise strategies in ECG interpretation as they advance in their medical careers. The main findings relate to how accurate each medical category is in ECG interpretation when considering their eye movements and visual behavior. The accuracy of the final ECG diagnosis was also associated with the expertise level of participants. Moreover, the increased level of participant expertise made experienced practitioners require less time to fixate on ECG abnormalities and decreased fixation counts, leading to correct diagnoses. Lastly, medical practitioners focus on certain ECG leads and specific waveform abnormalities according to their role in the health care sector and their expertise level.

#### Study Limitations and Future Works

Since eye-tracking data is idiosyncratic to every interpreter, a sample size of approximately 60 participants from different categories may not be representative enough. Sample size determination depends on what the designers of the study aim to represent. Recruited sample size may therefore vary according to the targeted population, CIs, and interpreters’ confidence level in their responses. Based on these uncontrollable factors, recruiting more participants and increasing the number of medical practitioner categories are necessary. We addressed this by recruiting a diverse and reasonable number of health care practitioners, but including larger numbers of participants in future work would contribute to a better understanding of visual expertise in ECG interpretation and understanding how different health care practitioners with different roles and expertise levels interpret ECGs. The richness of the study’s collected eye movement data has the potential to be further analyzed using machine learning algorithms to deeply reveal differences in visual behavior among the different categories of medical practitioners. We also plan on experimenting with more subtle examples of ECG diagnoses such as nonspecific/incomplete abnormalities and see how the experts deal with conflicting or vague data.

## Appendix

Multimedia Appendix 1 Multiple choice questions for the electrocardiogram eye-tracking experiment.

Multimedia Appendix 2 Sample grid-based areas of interest applied to the normal sinus rhythm electrocardiogram.

Multimedia Appendix 3 Definition of electrocardiogram samples used in the eye-tracking experiment.

Multimedia Appendix 4 Heat maps for all the categories of interpreters and all the electrocardiograms.

## Abbreviations

AOI: area of interest
ECG: electrocardiogram
