# Supplementary material for: Understanding Cardiology Practitioners’ Interpretations of Electrocardiograms: An Eye-Tracking Study
Source: JMIR Hum Factors. 2022 Feb 9;9(1):e34058. doi: 10.2196/34058 (PMC8867292; doi:10.2196/34058)
Supplement: Multimedia Appendix 1 [file humanfactors_v9i1e34058_app1.pdf]

Correct answer is highlighted in yellow:

1- What is the diagnosis? (image reference: personal collection)
A- Normal sinus rhythm
B- Ventricular tachycardia
C- Atrial fibrillation
D- Atrial tachycardia

2- What is the diagnosis? (image reference: https://thesgem.com/2015/10/sgem133-just-beat-it-atrial-fibrillation-with-diltiazem-or-metoprolol/)

A- Normal sinus rhythm
B- Ventricular tachycardia
C- Atrial fibrillation
D- Atrial flutter

3- What is the diagnosis? (image reference: https://jetem.org/hyperkalemia/)
A- ST elevation myocardial infarction
B- Hyperkalemia
C- Ventricular tachycardia
D- Left bundle branch block

4- What is the diagnosis? (image reference: personal collection)
A- Wolf-Parkinson-White syndrome
B- Ventricular tachycardia
C- Atrial fibrillation
D- Atrial flutter

5- What is the diagnosis? (image reference: personal collection)
A- Ventricular fibrillation
B- Ventricular tachycardia
C- Normal sinus rhythm
D- Atrial tachycardia

6- What is the diagnosis? (image reference: personal collection)
A- Brugada syndrome
B- Wolf-Parkinson-White syndrome
C- Long QT syndrome
D- Normal ECG

7- What is the diagnosis? (image reference: personal collection)
A- Ventricular paced rhythm
B- Right bundle branch block

C- Atrial paced rhythm
D- Hypercalcemia

8- What is the diagnosis? (image reference: personal collection)
A- Ventricular paced rhythm
B- Right bundle branch block
C- Ventricular tachycardia
D- Left bundle branch block

9- What is the diagnosis? (image reference: personal collection)
A- Ventricular paced rhythm
B- Non-ST elevation myocardial infarction
C- ST elevation myocardial infarction
D- Acute pericarditis

10- What is the diagnosis?
A- Sinus bradycardia
B- 1st degree AV block
C- Complete heart block
D- Ventricular tachycardia
