# Supplementary figures and images for: Understanding Cardiology Practitioners’ Interpretations of Electrocardiograms: An Eye-Tracking Study
Source: JMIR Hum Factors. 2022 Feb 9;9(1):e34058. doi: 10.2196/34058 (PMC8867292; doi:10.2196/34058)

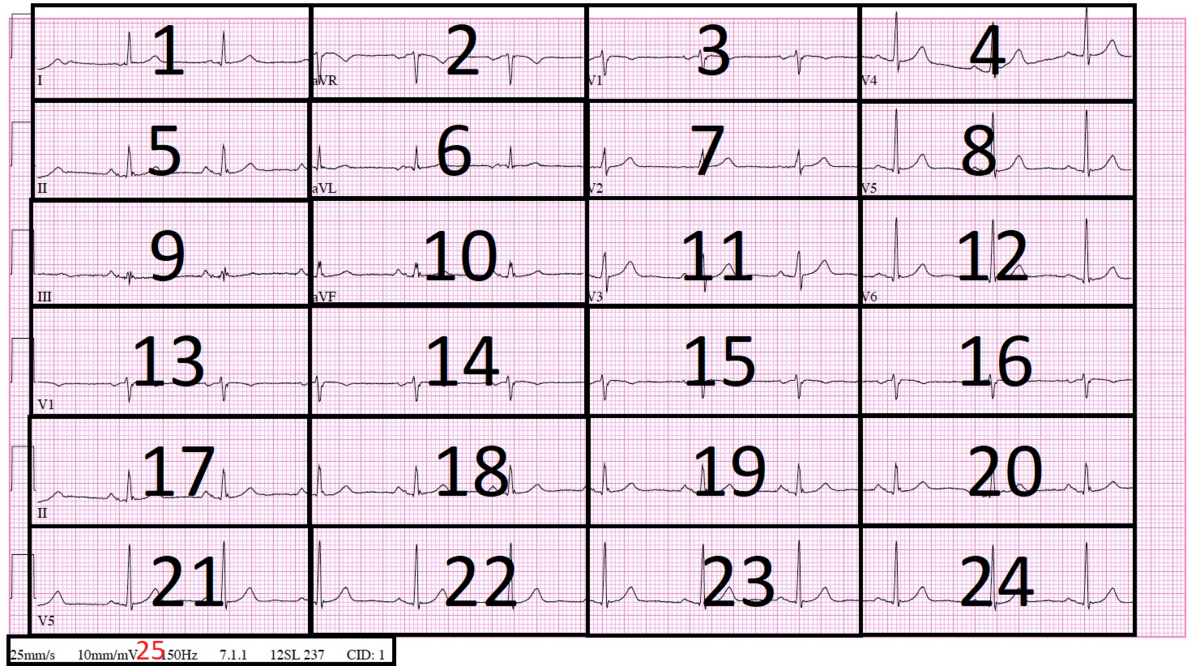

Supplement: Multimedia Appendix 2 [file humanfactors_v9i1e34058_app2.png]
