# Supplementary material for: Understanding Cardiology Practitioners’ Interpretations of Electrocardiograms: An Eye-Tracking Study
Source: JMIR Hum Factors. 2022 Feb 9;9(1):e34058. doi: 10.2196/34058 (PMC8867292; doi:10.2196/34058)
Supplement: Multimedia Appendix 3 [file humanfactors_v9i1e34058_app3.pdf]

ECG definitions:

ECG	Abbreviation	Definition
**Normal sinus rhythm**	NSR	A normal electrocardiogram rhythm
**Atrial fibrillation**	AFib or AF	Irregular, often rapid heart rate that commonly causes poor blood flow
**Hyperkalemia**	N/A	Potassium level imbalance in the blood (higher than normal)
**Atrial flutter**	N/A	An arrhythmia causing a rapid heart rate
**Ventricular tachycardia**	VT	Fast ventricular rate
**Wolff-Parkinson-White syndrome**	WPW	An accessory pathway between the atria and ventricles
**Ventricular paced rhythm**	N/A	An artificial pacemaker inserted to activate the ventricle(s)
**Left bundle branch block**	LBBB	Blockage of electrical impulse travelling down the left bundle branch
**ST-segment elevation myocardial infarction**	STEMI	A heart attack during which one of the major arteries is blocked. There are different locations of STEMI that look different on ECG: anterior STEMI, inferior STEMI, lateral STEMI, anterolateral STEMI, inferolateral STEMI
**Complete heart block**	AV block	A medical condition in which the nerve impulse generated in the sinoatrial node in the atrium of the heart cannot propagate to the ventricles
